# Application of artificial neural network in predicting EI

**DOI:** 10.37796/2211-8039.1029

**Published:** 2020-09-01

**Authors:** Elahe Allahyari

**Affiliations:** Social Determinants of Health Research Center, Faculty of Health, Department of Epidemiology and Biostatistics, Birjand University of Medical Sciences, Birjand, Iran

**Keywords:** Emotional intelligence, Artificial neural networks, Sociological variables

## Abstract

Emotional intelligence (EI) constitutes a whole set of non-cognitive capabilities, competencies, and skills that affect one's ability to deal successfully with environmental demands and pressures. Different factors such as gender, age, education, place of residence, etc. can influence this variable. Nevertheless, the influence of a multitude of factors involved in behavioral phenomena cannot often be controlled.

**Purpose:**

Therefore, some difficulty may often raise in finding associations between these variables using regression models as regression models are built on restrictive assumptions.

**Methods:**

In these cases, models such as artificial neural networks are excellent alternatives to regression models. In this study, the neural network model was used in SPSS software to predict the pattern held among the variables of age, gender, occupation, marital status, and education for predicting the EI of 901 individuals aged from 17 to 73 years.

**Results:**

The appropriate neural network model for EI prediction is a hyperbolic tangent transfer function with two neurons in the hidden layer and a sigmoid transfer function in the output layer. This network was able to predict EI in most of its dimensions with significant correlations and could demonstrate the neural network's advantage over regression models in predicting EI using sociological variables.

**Conclusion:**

This model is able to estimate the EI level in different occupational, educational, gender, and age groups, and provide the ground for planning to address potential deficiencies in each group.

## 1. Introduction

Over the past 50 years, attention has been directed more to the study of diseases and pathology than to the study of healthy humans [1]. Recently, however, with the new approach of positive psychology or perfect man, the previous perspective has changed. Scientists are now studying the positive aspects of human beings and finding ways whereby individuals can maximize their use of their talents and benefit from healthy mental states in life [1]. During the past few decades, human knowledge has gained valuable insights into the role of emotions in human life [2-4]. Researchers have found that an awareness of and the ability to control emotions can be linked with the success and happiness of individuals in all spheres of life. Therefore, emotional intelligence (EI) has been introduced as an integral part of education and learning, and its evaluation has been considered of particular importance in behavioral and psychometric sciences [5].

EI is the ability of individuals to perceive emotions and feelings in oneself and others and to respond appropriately to them, as well as to arouse, understand, regulate, and manage emotional responses. This ability is associated with one's understanding of him/herself and others, communication with others, and adaptation and compatibility with the environment, which are necessary to succeed in fulfilling social demands. It is, moreover, a tactical capability for personal performance [6]. Nonetheless, EI is one of those constructs that cannot be directly measured, and similar to the majority of constructs studied in behavioral and psychometrical sciences, a set of questionnaires are designed by Bar-On, Petrides, Salovey and Mayer, and Furnham, among/others [7-10]. The BarOn Emotional Quotient Inventory has become more popular because it makes the individual self-aware [7]. Moreover, it has been extensively used to evaluate and compare the EI of different groups and to establish the relationship between EI and other personality factors [11-13].

Since EI consists of a set of non-cognitive abilities, competences, and skills that rely on one's ability to succeed in coping with environmental demands and pressures, it may be associated with various factors including gender, age, education, and residence, among others. Numerous studies have compared and yielded conflicting results concerning EI in women and men [14–18]. Fomentez et al. found that the perceived quality of professional life was related to the perceptions and regulations of EI dimensions [19]. Studies have also shown that EI is a critical determinant of real-life outcomes such as success in high school and in health practices [20]. People with higher EI levels are more compatible with problems and more satisfied with life. Therefore, there is a significant relationship between EI and physical and mental health [7]. To assess the impact of different factors on EI, the majority of studies have utilized conventional models, including the regression models, or tried to match groups in order to control for confounding variables. However, most variables in behavioral research are not entirely controllable, and the influence of a multitude of factors involved in these phenomena is not usually controlled.

Today, new techniques known as intelligent systems are used to uncover intricate patterns among variables. The scientific branches relevant to intelligent systems include artificial life (AL), evolutionary strategies or genetic algorithm (GA), and artificial neural networks (ANN) [21]. In artificial life, optimization problems are solved by simulating the performance of living things and their behavior. In the evolutionary strategies branch, simulations of evolutionary theories (such as Darwin's theory) are applied to solve optimization problems. In artificial neural networks, as used in this study, the intelligence and the functioning of the brain are simulated, and the results are used to solve a variety of problems. In all these disciplines, the aim is to receive inspirations from problem-solving systems in nature and to apply them to solve scientific problems. Nevertheless, why do we rely on these systems despite the different statistical models available to recognize patterns? These systems have a memory, an ability to identify and learn, a parallel manner of functioning, and the power to generalize.

## 2. Purpose

Therefore, to predict and discover the relationships between variables in these systems, there is no need to establish restrictive assumptions such as equality of variances, normality of data distribution, and even linearity of the relationships. Moreover, the researcher does not need to find a similarity between the pattern found in the data and the already known functions [22]. Therefore, in this study, we aimed to determine the effect of the variables of age, gender, occupation, residence, marital status, and education on EI using the artificial neural network model and to determine the best network for predicting EI as per these variables.

## 3. Methods

This study was performed on Iranian men and women aged 17-73 years. For this purpose, we randomly selected a hospital, a university of medical sciences, and a shopping center in the cities of Birjand, Mashhad, and Shiraz. In each city, 110 staff members of the university from different departments, 110 hospital staff from different wards, and 110 individuals working in the market were selected from among those who were willing to participate in the study. At first, the research aim was described to the subjects whereby they signed informed consent forms for participation. Subjects were then asked to complete the BarOn emotional quotient inventory (EQ-i). The questionnaires were completed via interview for those who were not literate. The incomplete questionnaires were removed, and the data from the remaining 901 individuals were analyzed in SPSS-22 software using the artificial neural network method.

### 3.1. BarOn EQ inventory

In this study, Bar-on EQ inventory was used to measure EI. The BarOn EQ test was designed in five composite scales: intrapersonal functioning, inter-personal skills, adaptability, general mood, and stress management. The test consists of 133 items on 15 subscales, as presented in Table 1. The answers range from strongly disagree (1) to strongly agree (5) on a five-point Likert scale. However, a number of items are reversely scored as outlined in Table 1. The individual's EI score in each of the subscales, composite scales, and the overall score is obtained from the sum of the scores for the respective section. BarOn administered the questionnaire to 3,831 people from five countries (Argentina, Germany, India, Nigeria, and South Africa) of whom 48.8 percent were men and 51.2 percent were women [23]. He systematically standardized the inventory in North America, showing that the test had good validity and reliability. In 2013, Dehshiri assessed the validity and reliability of the inventory on 500 male and female students, aged 18 to 40 years, from different disciplines studying in Isfahan, Isfahan University of Medical Sciences, and Khorasgan Azad University. He reduced the number of items to 90 and reported a Cronbach's alpha coefficient of 0.93 [24].

### 3.2. Data analysis

Each neural network is generated by the inter-connection of neural model neurons. At the beginning of each input channel to a neuron, there is a numerical coefficient, which is multiplied by the stimulation intensity. The result is called a weighting input, which creates a stimulatory signal input on the neuron body, if it is positive, and an inhibitory signal input if it is negative. The amount of all of these inhibitory or input signals that reach the neuron body from different inputs is linearly summed. If this sum is lower than the threshold, the nerve cell will remain silent. Otherwise, the neuron is activated and generates a constant current in the output or outputs. In practice, the mathematical function of the neuron body can be a sigmoid, hyperbolic tangent, linear, or any other function [25].

Neural network learning may use supervised, unsupervised, and reinforced learning [26]. In supervised learning, which is used in this study, the desired results are presented to the network by a supervisor and the network adjusts the neural network weights based on the error between the estimated outputs and the expected outputs. The arrangement of nodes and the way they are connected in different layers of the network is called network topology [27]. The network topology used in this study is multilayer normal feedforward, which is used in 90 to 95 percent of cases [28, 29]. In this method, each layer is the input vector for the next layer and the output vector for the previous layer. The last layer in this sequence will be the response variable.

In order to determined the impact of age, sex, education, marital status, occupation, and place of residence on the five composite scales of EI, this study used a supervised neural network model. For this purpose, the data are randomly divided into training (70%) and testing (30%) sets. Subsequently, neural networks with one and two hidden layers were used to design the model. The number of neurons in the hidden layer varies from 2 to 4, and a combination of different functions will be used for the hidden (sigmoid and hyperbolic tangent), and output (sigmoid, hyperbolic tangent, and linear) layers. One consecutive step with no decrease in error was stopping criteria used in this study and standardized data were used in order to overcome the scale dependent variables. And, the neural network with the fewest sum of square errors in both training and testing sets was selected as the optimization algorithm. Finally, the impact of the factors will be determined in selected algorithm and correlation of predicted and observed values will be reported in optimized ANN algorithm to demonstrate the advantages of the ANN model.

## 4. Results

As Table 2 indicates, 901 people were studied whom 700 were men and 201 were women. The majority were married (82%) and residing in urban areas (89.5%). The frequency rates of people involved in managerial, professional, service, sales, administrative, agricultural, construction, installation, production, and transportation sectors were 9, 13.3, 11.3, 11.1, 20.9, 3.6, 9.5, 9.3, 5.1, and 6.9 percent. Most of the participants had a high school diploma or a bachelor's degree, and their mean age was 35.37 ± 9.33 years.

**Fig. 1:**
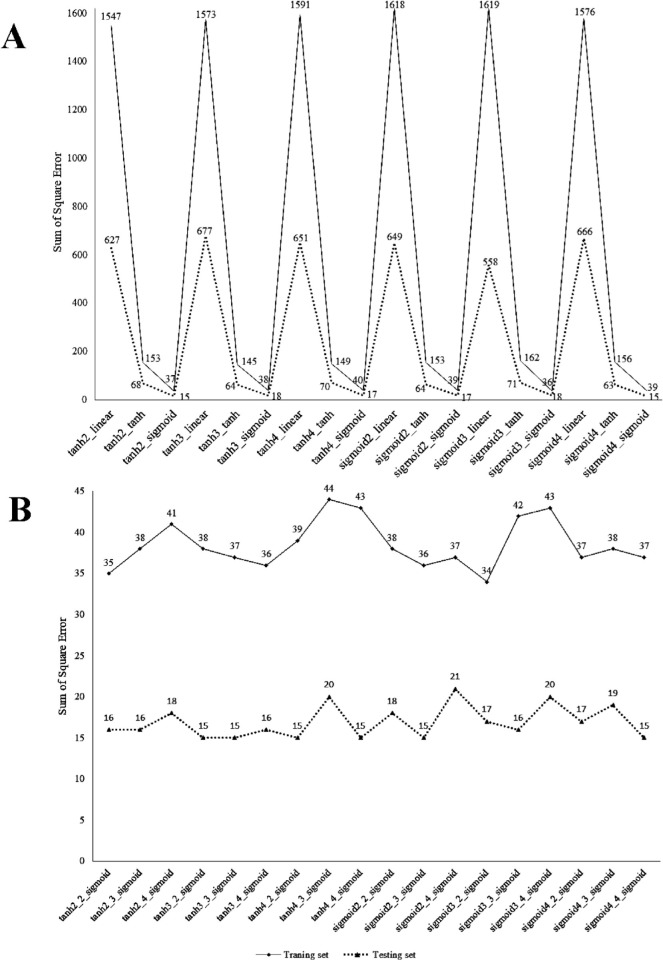
A)The sum of square error of ANN models for different transfer functions; B) The sum of square error of ANN models for 2 hidden layers.

To select the appropriate function in the neural network, we first evaluated all neural network combinations with 2, 3, and 4 neurons and sigmoid and hyperbolic tangent functions in the hidden layer, as well as hyperbolic, linear, and sigmoid tangent functions in the output layer. Fig. 1A depicts the error sum of squares in the training and testing groups for these neural networks. As can be seen, two sets of neural networks have the lowest error rate, including a net with a hyperbolic tangent function in the hidden and a sigmoid function in output layers and a net with sigmoid functions in the both hidden and output layers.

Nevertheless, among other factors affecting the performance of the neural network is the number of hidden layers. To asses this, all neural networks characterized by two intermediate layers, 2, 3, and 4 neurons, and selected functions (i.e., hyperbolic tangent function in the hidden and sigmoid function in the output layers and sigmoid function in both the hidden and output layers) were evaluated (Fig. 1B). A comparison of the results of Fig. 1A and Fig. 1B shows that the increasing number of hidden layers could not improve the fitting of the network significantly. Therefore, a neural network with hyperbolic tangent functioning hidden layer with two neurons, and a sigmoid function in the output layer was chosen as the optimal neural network. The optimal network was used to predict people's EI using the variables of age, gender, occupational sector, place of residence, education, and marital status. The error sums of squares in the training and testing groups for this network were 37 and 15, respectively, indicating that the model did not overfit in the training group given the selection of 620 individuals in the training group and 281 in the testing group (Fig. 1).

**Fig. 2:**
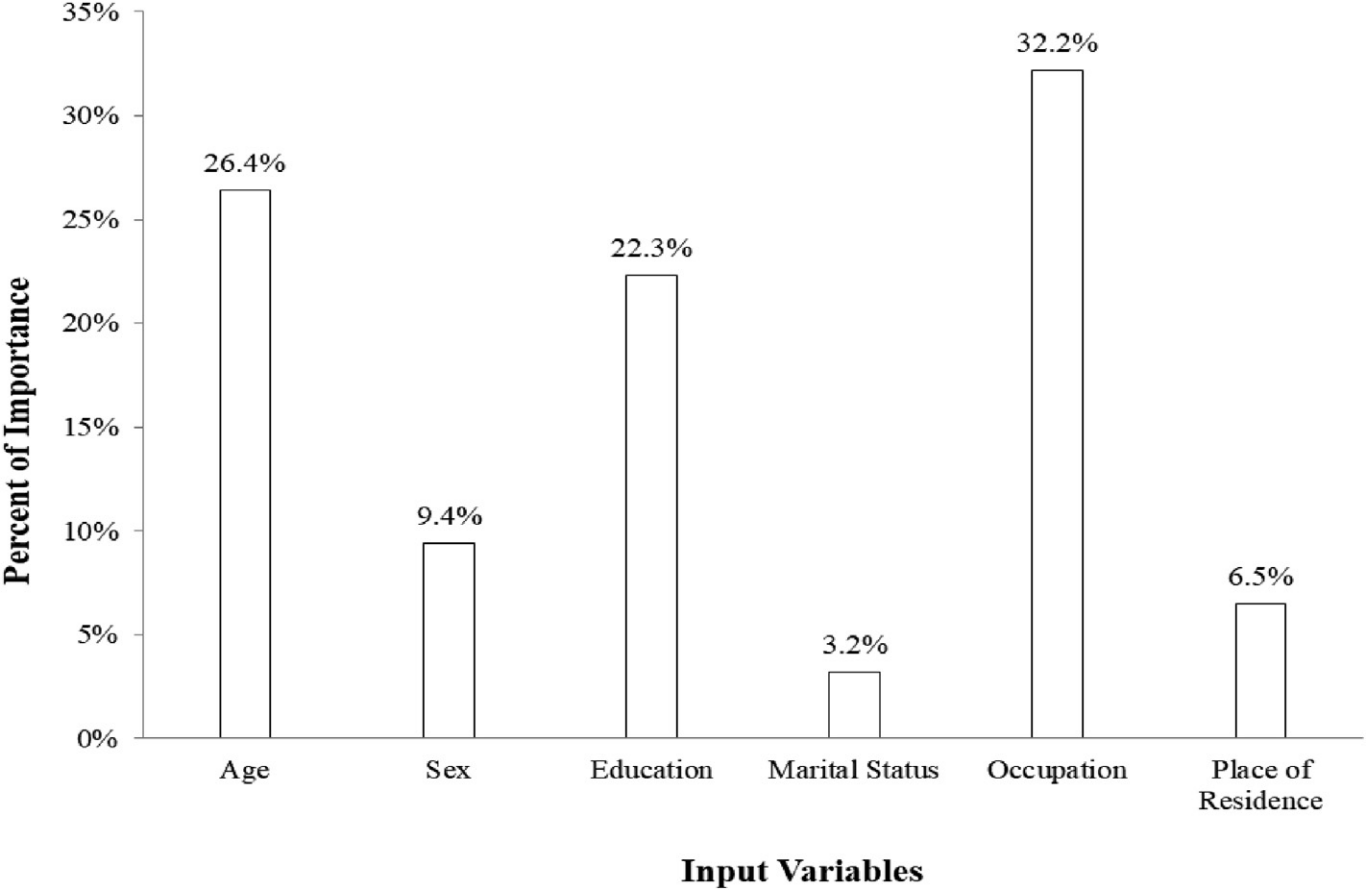
The variable importance from the selected Artificial Neural Network.

There was a significant correlation between the estimated values and the actual values in the composite scales of intrapersonal functions, adaptability, and stress management (Table 3). Therefore, in these scales, the neural network model has been able to predict the EI of individuals very well. Fig. 2 can be used to determine the importance of variables studied in the prediction of EI scales. This figure clearly reveals that in the optimal neural network, occupational sector, age, education, gender, residence, and marital status are, respectively, the most significant factors in predicting people's EI in the five scales.

## 5. Discussion

Constructs such as EI widely affect an individual's performance and improvement of his/her life [30-32]. Therefore, to recognize the factors affecting EI, to determine the importance of each of these factors, and to provide a prediction model of EI can be a vital contributor to the promotion of both people's lives and the society in large. In this study, an artificial neural network could develop a suitable model for predicting EI of individuals according to their sociological factors. A number of these factors with a significant impact on EI, such as the education level, can be promoted [33, 34]. It is even possible to educate those in specific gender groups and improve living conditions in rural areas. In addition, the optimal neural network can be used to estimate the EI level in each of the occupational sectors and to plan for any potential deficiencies in each group.

Although we could not find a study to have built on artificial neural networks to investigate the effect of sociological factors on EI, several studies were found to have used regression models or group comparisons for this purpose. Some of these studies estimated women's EI as higher, some considered men's EI as higher, and some rejected any relationship [35-37]. Concerning the association between age and EI, the results were similarly inconsistent, with some believing that EI increased with age, while others reported a reverse trend [38, 39]. Some studies also used regression models to assess the simultaneous impact of several factors on EI. Ghazizadeh et al. maintained that the variables of marital status, gender, and age are not conducive to the EI of MS patients and medical residents, while EI in MS patients increased with education [38-41]. Harwood et al. also found no significant impact of age and place of residence on EI, although they rated gender as significant, acknowledging that these variables could only account for 0.148 of the changes in EI [42]. Comparing the results of the present study with studies mentioned above, we can understand the appropriate performance of neural network models in cases such as this study where the effects of variables such as age, gender, education, etc., may have interactions with each other in predicting people's EI. However, it should be remembered that in our study, sociological factors could not predict the general mood of individuals. Therefore, further studies are recommended that include other factors, such as family conditions and personal talents, which can influence the EI.

## Figures and Tables

**Table 1:** items of 15 subscales of BarOn emotional quotient inventory.

EI composite scales	EI subscales	Items
intrapersonal functioning	Emotional self-awareness	6, 21^a^, 36^a^, 51, 66, 81^a^
	Assertiveness	15^a^, 30, 45^a^, 60, 75^a^, 90^a^
	Self-regard	10, 25, 40^a^, 55, 70, 85
	Self-actualization	5, 20^a^, 35^a^, 50^a^, 65, 80^a^
	Independence	3, 18^a^, 33^a^, 48^a^, 63^a^, 78^a^
interpersonal skills	Empathy	14, 29, 44, 59, 74, 89
	Interpersonal relationships	8, 23, 38, 53, 68, 83
	Social responsibility	13, 28, 43, 58^a^,73, 88
adaptability	Problem solving	1, 16, 31, 46, 61^a^, 76
	Reality testing	77, 22^a^, 37^a^, 52^a^, 67^a^, 82^a^
	Flexibility	12^a^, 27^a^, 42, 57, 72^a^, 87^a^
general mood	Happiness	2^a^, 17^a^, 32, 47,62, 77^a^
	Optimism	9, 24, 39, 54, 69, 84^a^
stress management	Stress tolerance	4, 19, 34^a^, 49, 64^a^, 79^a^
	Impulse control	11^a^, 26^a^, 41^a^, 56^a^, 71^a^, 86^a^

a Items are reversely scored.

**Table 2:** demographic characters in study population.

Variable	N (%)
Sex	
Male	700 (77.7%)
Female	201 (22.3%)
Education	
Primary or secondary school	146 (16.2%)
High School Diploma	273 (30.3%)
Associate Degree	116 (12.9%)
Bachelor	292 (32.4%)
Master	64 (7.1%)
P.h.D	10 (1.1%)
Marital Status	
Single	162 (18%)
Married	739 (82%)
Occupation sectors	
Managerial	81 (9%)
Professional	120 (13.3%)
Service	102 (11.3%)
Sales	100 (11.1%)
Administrative	188 (20.9%)
Agricultural	32 (3.6%)
Construction	86 (9.5%)
Installation	84 (9.3%)
Production	46 (5.1%)
Transportation	62 (6.9%)
Place of Residence	
City	806 (89.5%)
Village	95 (10.5%)

**Table 3:** correlation between the estimated values of ANN model and the actual values in five composite scales of EI.

estimated actual	1	2	3	4	5
1	0.151 (P < 0.001*)				
2		0.046 (P = 0.167)			
3			0.117 (P < 0.001*)		
4				0.000 (P = 0.990)	
5					0.110 (P = 0.001*)

1 intrapersonal functioning,

2 interpersonal skills,

3 adaptability,

4 general mood,

5 stress management.

* Significant correlation at *α* = 0.05.
